# Isoliquiritigenin Derivative Regulates miR-374a/BAX Axis to Suppress Triple-Negative Breast Cancer Tumorigenesis and Development

**DOI:** 10.3389/fphar.2020.00378

**Published:** 2020-03-31

**Authors:** Fu Peng, Liang Xiong, Xiaofang Xie, Hailin Tang, Ruizhen Huang, Cheng Peng

**Affiliations:** ^1^Key Laboratory of Drug-Targeting and Drug Delivery System of the Education Ministry and Sichuan Province, West China School of Pharmacy, Sichuan University, Chengdu, China; ^2^Key Laboratory of Systematic Research of Distinctive Chinese Medicine Resources in Southwest China, Chengdu University of Traditional Chinese Medicine, Chengdu, China; ^3^Cardiovascular Department, Hospital of Chengdu University of Traditional Chinese Medicine, Chengdu, China; ^4^Department of Breast Oncology, Sun Yat-Sen University Cancer Center, State Key Laboratory of Oncology in South China, Guangzhou, China

**Keywords:** 3′,4′,5′,4″-tetramethoxychalcone, triple-negative breast cancer, apoptosis, miR-374a, Bax

## Abstract

Triple-negative breast cancer (TNBC) is a subtype of breast cancer that accounts for the largest proportion of breast cancer-related deaths. Thus, it is imperative to search for novel drug candidates with potent anti-TNBC effects. Recent studies suggest that isoliquiritigenin (ISL) can significantly suppress the growth, migration, and invasion of breast cancer cells. We previously synthesized ISL derivatives and found that 3′,4′,5′,4″-tetramethoxychalcone (TMC) inhibits TNBC cell proliferation to a greater degree than ISL. The present study aimed to investigate the mechanisms underlying the anti-TNBC effects of TMC *in vitro* and *in vivo*. We show that TMC significantly inhibits the proliferative, migratory, and invasive abilities of MDA-MB-231 and BT549 cells. TMC induces apoptosis through the upregulation of Bax and downregulation of Bcl-2. PCR arrays demonstrate a significant decrease in miR-374a expression in TNBC cells after 24-h TMC treatment. MiR-374a is overexpressed in TNBC cells and has oncogenic properties. Real-time PCR analysis confirmed that TMC inhibits miR-374a in a dose-dependent manner, and luciferase assays confirmed that BAX is targeted by miR-374a. Further, we show that TMC increases Bax protein and mRNA levels by inhibiting miR-374a. TMC also attenuates TNBC tumor volumes and weights *in vivo*. These results demonstrate that TMC inhibits TNBC cell proliferation, foci formation, migration, invasion, and tumorigenesis, suggesting its potential to serve as a novel drug for treating TNBC through miR-374a repression.

## Introduction

Breast cancer is the cancer most commonly diagnosed in females worldwide (Siegel et al., 2018), and part of its devastating impact is due to the existence of triple-negative breast cancer (TNBC) (Choi et al., 2019). TNBC is a subtype of breast cancer that is hormone-receptor negative and human epidermal growth factor receptor 2-negative (Perou et al., 2000), accounting for 15% to 20% of the global incidence of breast cancer (Mayer et al., 2014). Compared to other cancer subtypes, current targeted or endocrine therapies cannot treat TNBC, leading to its incidence being disproportionally associated with breast cancer-related deaths (Bianchini et al., 2016). Thus, it is imperative to search for alternative TNBC therapeutics with novel molecular targets.

MicroRNAs (miRNAs) are a class of small non-coding RNAs that negatively regulate their target genes through direct binding to the 3′ untranslated region (3′UTR) region of the targets (Peng et al., 2016b). Emerging evidence suggests that miRNAs play significant roles in the development and progression of cancers (Lin and Gregory, 2015) including breast cancer. MiR-374a is commonly expressed at high levels in various types of cancer (Miko et al., 2009; Huang et al., 2011; Namlos et al., 2012). The oncogenic abilities of miR-374a were first reported in non-small cell lung cancer (NSCLC), where it promoted cell migration and invasion (Miko et al., 2009; Wang et al., 2014). Additionally, miR-374a promotes cell proliferation, cell migration, cell invasion, and metastasis in hepatocellular carcinoma (Zhao et al., 2014; Liu et al., 2016). MiR-374a also promotes cell growth in gastric cancer through its direct interaction with SRCIN1 (Xu et al., 2015). Recent studies suggest that miR-374a can promote chemoresistance in gastric cancer (Ji et al., 2019). In breast cancer, miR-374a increases the percentage of migratory and invasive breast cancer cells and promotes metastasis through the Wnt and Akt pathways (Cai et al., 2013). Accordingly, miR-374a knockdown inhibits breast cancer cell proliferation, colony formation, migration, and invasion (Zhang et al., 2018). Additional studies indicate that miR-374a is upregulated in TNBC patients, and high expression of miR-374a promotes TNBC development (Son et al., 2019).

**Figure 1:**
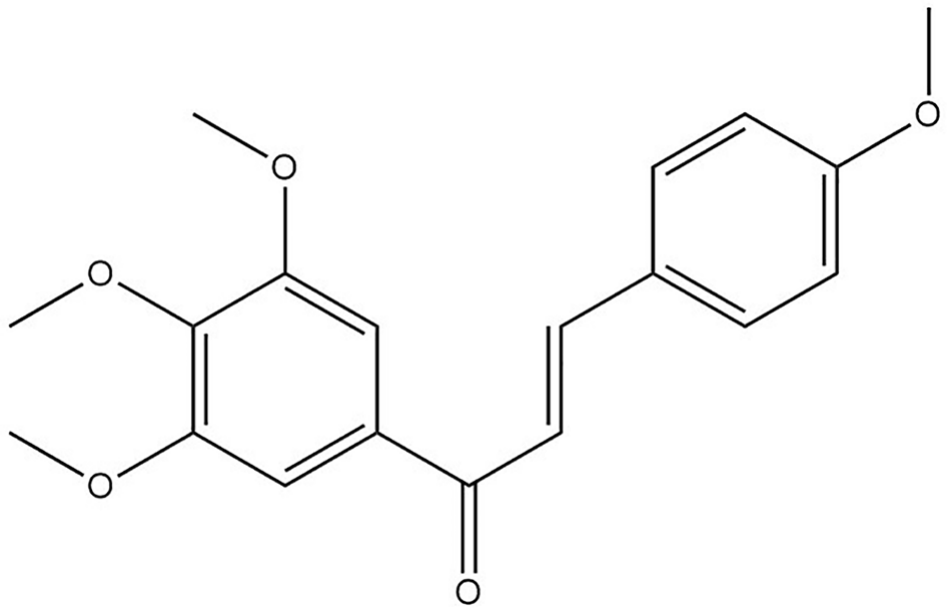
The chemical structure of tetramethoxychalcone (TMC) constructed using ChemBioDraw.

In recent times, Chinese medicines have been gaining acceptance as potent anticancer agents, with dramatic inhibitory effects on cancer cell growth and movement (Jun et al., 2018; Lei et al., 2019). *Spatholobus suberectus* Dunn (*S. suberectus*), a common Chinese herb used for treating blood-stasis related diseases, exerted potential anticancer effects (Kang et al., 2003; Kim et al., 2018; Lim et al., 2019). Especially, *S. suberectus* and flavonoids from *S. suberectus*, displayed a potent inhibitory effect on breast cancer (Peng et al., 2019b). *S. suberectus* could inhibit breast cancer cells migration through PI3K/AKT pathway and suppress breast cancer cells proliferation through lactate dehydrogenase (Wang et al., 2013b; Sun et al., 2016). Isoliquiritigenin (ISL), a natural flavonoid extracted from *S. suberectus*, exerts potent anti-cancer effects during breast cancer development (Lorusso and Marech, 2013; Wang et al., 2013a). In a previous study, we modified the ISL structure, synthesized its derivatives and found 3′,4′,5′,4″-tetramethoxychalcone (TMC) (**Figure 1**, **Table S1**) had a greater inhibitory effect on breast cancer cell viability than ISL, especially on MDA-MB-231 cells (Peng et al., 2016a). Further study found that it had a better anti-breast cancer effect on TNBC cells than cisplatin (**Figure S1**). Thus, the mechanisms underlying the effect of TMC on TNBC migration, invasion, and growth deserve further investigation.

In the present study, we find that TMC inhibits TNBC proliferation through the induction of apoptosis and suppresses TNBC migration and invasion. TMC also inhibits TNBC foci formation and tumor growth. PCR arrays reveals that miR-374a is dramatically downregulated in response to 24h TMC treatment. MiR-374a directly targets BAX as revealed by luciferase assays. Additionally, miR-374a overexpression reverses the proapoptotic effects of TMC in TNBC. Taken together, our results indicate that TMC can be considered as a novel TNBC drug candidate due to its ability to regulate miR-374a expression.

## Materials and Methods

### Chemicals and Reagents

TMC was prepared as previously described (referred as compound 3 h) (Peng et al., 2016a), and its purity was >98%. Xylene, eosin Y, hematoxylin, and other commonly used chemicals were purchased from Sigma (St. Louis, MO). Primary and secondary antibodies were obtained from Cell Signaling Technology (Danvers, MA). ECL Advance reagent was purchased from Merck-Millipore (St. Louis, MO). RNAiso Plus reagent, SYBR Green master mix, and the PrimeScript RT Reagent Kit with gDNA Eraser were obtained from TaKaRa (Bio Inc., Shiga, JP). The MiRNeasy Mini Kit was obtained from Qiagen (Hilden, DE) and the TaqMan MicroRNA Reverse Transcription Kit was obtained from Ambion (Life Technologies, MA, USA).

### Cell Culture

MDA-MB-231, BT549, MCF-10A, 4T1, 293T, and MCF-7 cells were incubated at 37°C in a 5% CO_2_ incubator purchased from American Type Culture Collection (ATCC, USA). MDA-MB-231, BT549 and 4T1 cells are TNBC cell lines. MCF-7 cells are hormone-receptor positive breast cancer cells. MCF-10A cells are normal human mammary epithelial cells. All growth mediums were supplemented with 10% fetal bovine serum (FBS), 1% penicillin, and 1% streptomycin from Gibico (Life Technologies, MA, USA), except for MCF-10A cells. MCF-10A cells were cultured in special keratinocyte serum-free medium. MDA-MB-231, BT549, and 293T cells were cultured in DMEM, while 4T1 and MCF-7 cells were cultured in RPMI 1640 medium.

### CCK-8 Assay

MDA-MB-231 and BT549 cells were seeded in 96-well plates at a density of 5 × 10^3^ cells/well. Two breast cancer cell lines were exposed to TMC for 24 h and 48 h. Cell viability was assessed using the CCK-8 kit obtained from MedChemExpress (NJ, USA) according to the manufacturer’s instructions. After TMC treatment, the cells were treated with 10 μl of the reagent for 2 h in a 5% CO_2_ incubator, and the absorbance at 450 nm was determined by enzyme-linked immunosorbent assay (ELISA) on a plate reader. These experiments were performed in triplicate.

### Foci Formation Assay

Foci formation assays were performed as previously described (Tang et al., 2018). Briefly, MDA-MB-231 and BT549 cells were seeded at a density of 1 × 10^3^ cells in six-well plates. Clones were stained with 0.5% crystal violet, and foci numbers were counted. The experiments were performed in triplicate.

### Wound Scratch Assay

MDA-MB-231 and BT-549 cells were seeded at a density of 3 × 10^5^ cells/ml into Culture-Inserts (Ibidi GmbH, Martinsried, DE) according to the manufacturer’s instructions. Images were recorded at the beginning and end of the experiment using a Zeiss Axio Lab A1 microscope (Oberkochen, DE). These experiments were performed in triplicate.

### Chamber Migration and Invasion Assays

Chamber migration and invasion assays were performed for according to the instructions from Corning Inc (NY, USA). MDA-MB-231, and BT549 cells were seeded on the upper surface at a density of 1.25 × 10^5^. After TMC treatment, migrating and invading cells were fixed with 4% PFA before 0.5% crystal violet staining. The remaining cells were imaged using a Zeiss Axio Lab A1 microscope and counted in triplicate in different fields of view.

### Flow Cytometric Analysis

The apoptosis analysis kit, equipment, and software were purchased from BD Company (CA, USA). MDA-MB-231 and BT549 cells were seeded in 6-well plates at a density of 5 × 10^5^ cells/well. The percentage of apoptotic cells after 24-h TMC treatment was detected using the Annexin V/PI staining apoptosis analysis kit according to the manufacturer’s instructions. Stained samples were analyzed by FACSAria SORP within 1 h. The experiments were performed in triplicate.

### Immunoblotting

Protein extraction was performed using the Cell lysis buffer from Sigma. Protein (20 μg) was resolved on 10% SDS-PAGE gels and transferred onto PVDF membranes from Merck-Millipore (St. Louis, MO). After blocking with 5% bovine serum albumin (BSA), the membranes were incubated with primary antibodies against Bax, Bcl-2, and β-actin (loading control) at 4°C overnight. After probing with secondary antibody, Western blots were developed using ECL Advance reagent and evaluated using Image Lab Software from Bio-Rad (Kidlington, UK).

### Quantitative Real-Time PCR

Total RNA was extracted using the RNAiso Plus reagent. Reverse-transcription of mRNA was performed using the PrimeScript RT Reagent Kit with gDNA Eraser, and real-time PCR experiments were performed using the SYBR Green master mix. GAPDH was selected as the loading control for mRNA expression analyses. miRNAs were extracted using the miRNeasy Mini Kit. miRNA reverse-transcription was conducted using the TaqMan MicroRNA Reverse Transcription Kit, and real-time PCR experiments were performed using the SYBR Green master mix. U6 was selected as the loading control for miRNA expression analyses. The corresponding primers for detecting BAX, GAPDH and U6 are listed in **Table S2**.

### Xenograft Tumor Growth Assays

MDA-MB-231 cells (2 × 10^6^) were inoculated into mammary gland fatpads of 4-week-old nude mice. After successful modeling, nude mice were randomly divided (*n* = 5) into a control group, low dose group (TMC, 20 mg/kg/d) and high dose group (TMC, 40 mg/kg/d). Twenty-one days after the intraperitoneal injection of TMC, the mice were euthanized and the tumor weights and volumes were measured. All animal experiments were performed according to the standard institutional guidelines of Sichuan University and Chengdu University of Traditional Chinese Medicine.

### PCR Array

MiRNA extraction, reverse transcription, and real-time PCR reactions were performed as previously described. PCR arrays were performed using the miRNA miRNome PCR Panel from Qiagen according to the manufacturer’s instructions.

### Luciferase Reporter Assay

The pMIR-REPOR miRNA Expression Reporter Vector System was used. We inserted the miR-374a-binding sequence of BAX (with or without mutation) to mimic the regulation of BAX by miR-374a through its 3′UTR. The primers used in these experiments are listed in **Table S3**. Dual-Glo Luciferase assays were performed according to the instructions from Promega (Madison, WI). The *Renilla* luciferase activity and firefly luciferase activity were recorded using a SpectraMax M5 (MD, USA).

### Cell Transfection

MirVana miRNA-374a mimic and mirVana miRNA Mimic Negative Control were obtained from Ambion. Lipofectamine 2000 Transfection Reagent was purchased from Invitrogen (Life Technologies, MA, USA). Transfections were performed according to the manufacturer’s instructions. MiR-374a mimic or negative control were mixed with diluted Lipofectamine 2000 Transfection Reagent as 1:1 ratio and incubated for 15 min at room temperature. MiRNA-lipid complexes were added to MDA-MB-231 and BT549 cells (60%–80% coverage) on the plate. The cells were incubated at 37°C in a 5% CO_2_ incubator for 24 h.

### Data Analysis

Statistical analysis was performed with GraphPad Prism 7.0 (USA). Two-tailed Student’s *t*-tests and one-way ANOVAs were used to evaluate statistical significance (*p < 0.05, **p < 0.01).

## Results

### TMC Suppresses TNBC Cell Viability and Foci Formation

**Figure 2:**
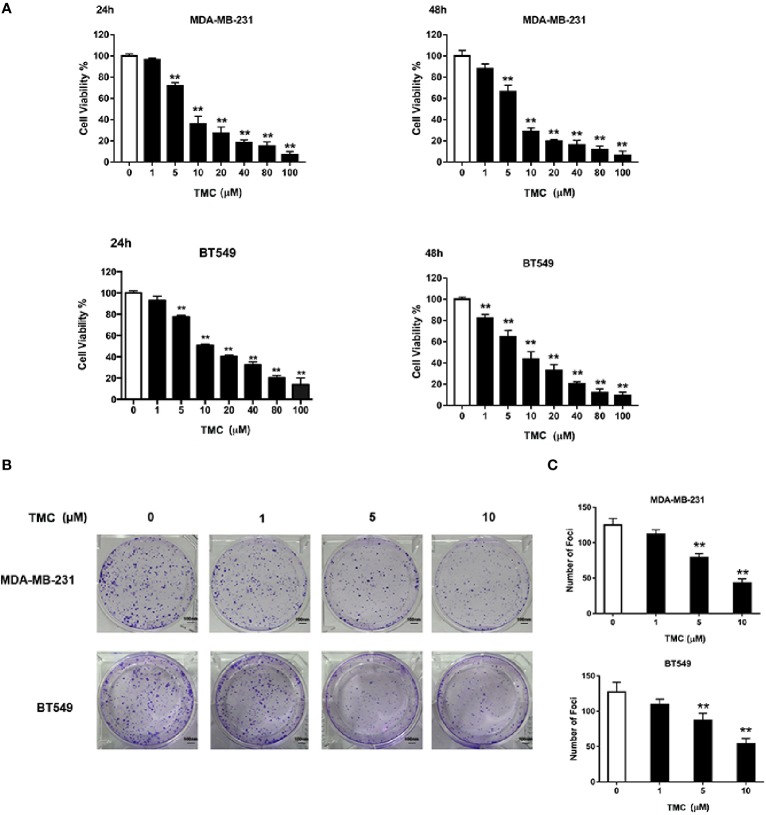
TMC inhibits triple-negative breast cancer (TNBC) cell proliferation. **(A)** Cell viability of MDA-MB-231 and BT549 cells after exposure to TMC for 24 and 48 h. **(B)** Representative images and rate of foci formation in MDA-MB-231 and BT549 cells after 24-h treatment. **(C)** Number of foci in MDA-MB-231 and BT549 after TMC 24-h treatment.

We used CCK-8 assays to determine the effects of varying TMC concentrations (1, 5, 10, 20, 40, 80, and 100 μM) on MDA-MB-231 and BT549 cells (**Figure 2A**). The results suggested that TMC (> 5 μM) significantly inhibited TNBC cell proliferation after treatment for 24 and 48 h (*p* < 0.01). The IC_50_ values of 24-h TMC treatment of MDA-MB-231 and BT549 cells were 8.696 and 14.28 μM, respectively. The cytotoxic effect of TMC was further investigated through foci formation assays. As shown in **Figures 2B, C**, TMC also suppressed the foci forming ability of MDA-MB-231 and BT549 cells after 24-h treatment. These results suggest that TMC has an inhibitory effect on TNBC proliferation.

### TMC Inhibits TNBC Migration and Invasion

**Figure 3:**
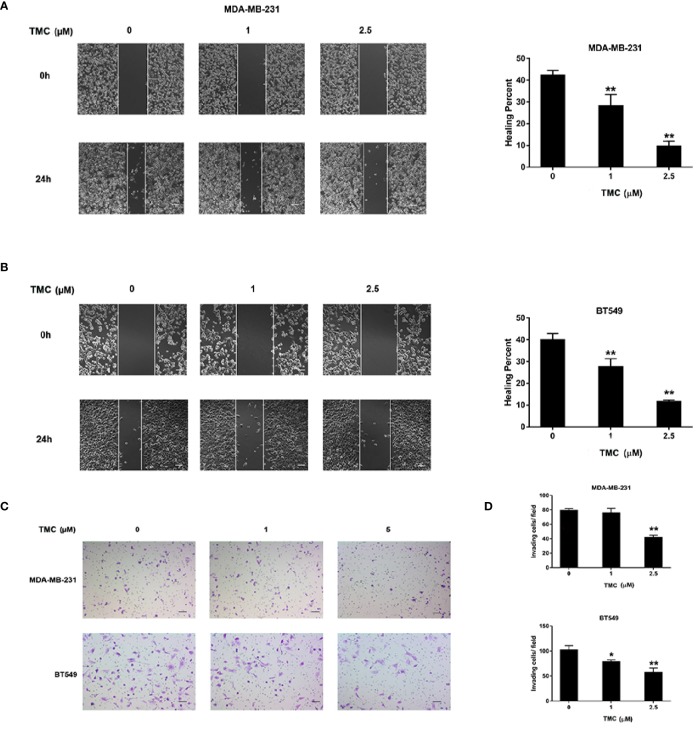
TMC suppresses TNBC cell migration and invasion. **(A)** Representative images and percentages of wound healing in MDA-MB-231 cells after exposure to TMC for 24 h. **(B)** Representative images and percentages of wound healing in BT549 cells after exposure to TMC for 24 h. **(C)** Representative images of chamber invasion assays in MDA-MB-231 and BT549 cells. **(D)** Percentages of invading cells in MDA-MB-231 and BT549 cells after exposure to TMC for 24 h. Compared with the control group (0 μM), *p < 0.05,**p < 0.01.

The effect of TMC on TNBC cell movement was determined by wound healing and chamber invasion assays. Results demonstrated that 24-h TMC treatment (> 1 μM) critically suppressed wound closure in MDA-MB-231 and BT549 cells, and the inhibitory effect increased with increasing drug concentration (*p* < 0.01) (**Figures 3A, B**). Chamber invasion assays demonstrated that MDA-MB-231 and BT549 cells had obvious invasive capacities, and 24-h TMC treatment reduced the number of invading cells (**Figures 3C, D**). TMC (2.5 μM) significantly inhibited the invasive ability of TNBC cells, however its suppressive effect was more mild in MDA-MB-231 cells than BT549 cells (*p* < 0.01). Taken together, these results indicate that TMC significantly inhibits the migratory and invasive capacities of MDA-MB-231 and BT549 cells.

### TMC Induces Apoptosis in TNBC Cells

**Figure 4:**
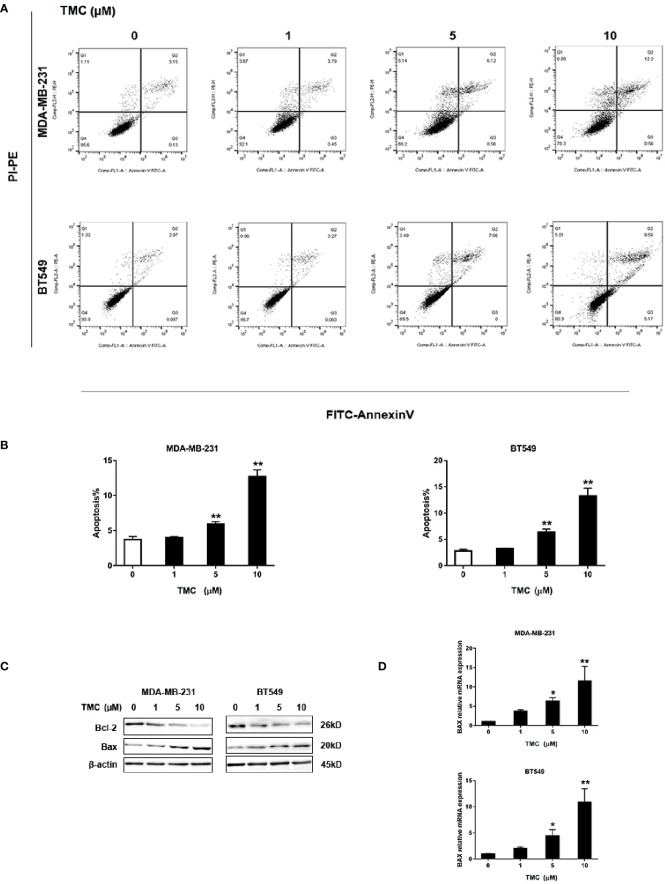
TMC increases the percentage of apoptotic cells in TNBC. **(A)** Representative images of apoptosis in MDA-MB-231 and BT549 cells determined by flow cytometry analysis after exposure to TMC for 24 h. **(B)** Percentages of apoptotic cells in MDA-MB-231 and BT549 cells after exposure to TMC for 24 h. **(C)** Western blot analysis of Bax and Bcl-2 after exposure to TMC for 24 h. **(D)** Real-time PCR analysis of BAX expression after exposure to TMC for 24 h. Compared with the control group (0 μM), *p < 0.05,**p < 0.01.

We utilized the Annexin V/PI staining apoptosis analysis kit TMC to quantify apoptotic cells. The results demonstrated that 24-h TMC treatment (> 5 μM) increased the percentage of apoptotic MDA-MB-231 and BT549 cells (*p* < 0.01) (**Figures 4A, B**). The proapoptotic effect of TMC was enhanced with increased drug dosage. Further, Western blot analyses demonstrated that 24-h TMC treatment promoted Bax expression and repressed Bcl-2 expression in a dose-dependent manner in TNBC cells (**Figure 4C**). Bax is a proapoptotic factor that promotes mitochondrial apoptosis. BCL-2 is an anti-apoptotic factor that inhibits proapoptotic proteins and promotes cellular survival. Real-time PCR confirmed that TMC increased Bax mRNA levels in a dose-dependent manner (*p* < 0.01) (**Figure 4D**). Collectively, these findings suggest that TMC triggers apoptosis in MDA-MB-231 and BT549 cells by increasing Bax and decreasing Bcl-2 expression.

### TMC Restrains TNBC Tumor Formation

**Figure 5:**
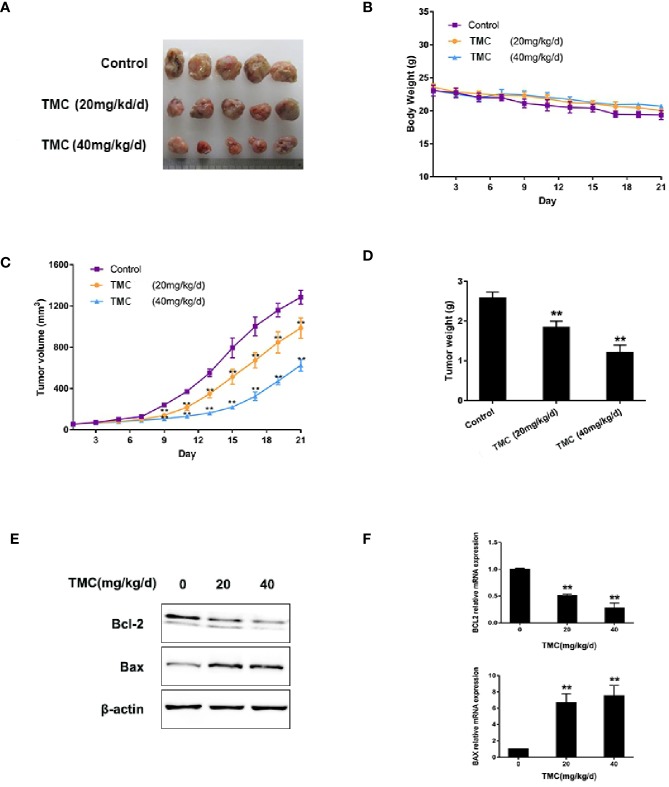
TMC represses TNBC tumorigenesis. **(A)** Tumor tissues collected at the end point. **(B)** Mice weights measured during the experiment. **(C)** Tumor volumes measured during the experiment. **(D)** Tumor weights at the end point. **(E)** Western blot analysis of Bax and Bcl-2 after TMC administration. **(F)** Real-time PCR analysis of BAX and BCL2 after TMC administration. Compared with the control group (0 μM), **p < 0.01.

The *in vivo* anti-cancer effect of TMC was investigated using MDA-MB-231 xenografts. TMC was administered to nude mice through intraperitoneal injection at 20 mg/kg/d (low dose group) and 40 mg/kg/d (high dose group) for 21 days. The mice weights and tumor volumes were recorded every three days. Tumor volumes were calculated according to a standard formula: (mm^3^) = L × W^2^/2. The tumors and other corresponding tissues were collected and measured after sacrificing the mice. Results exerted that TMC drastically suppressed TNBC tumor volumes in both the low dose and high dose groups (*p* < 0.01) (**Figures 5A, C**). In addition, TMC administration significantly reduced tumor weights after 21-day treatment (*p* < 0.01) (**Figure 5D**). Mouse weight did not decrease dramatically after TMC administration, even in the high dose group (**Figure 5B**). TMA could increase Bax protein and mRNA levels, and decrease Bcl-2 protein and mRNA levels *in vivo* (**Figures 5E, F**). Altogether, these findings suggest that TMC significantly inhibits TNBC tumor growth without obvious cytotoxic effects on normal tissues.

### TMC Downregulates Highly Expressed miR-374a in TNBC Cells

**Figure 6:**
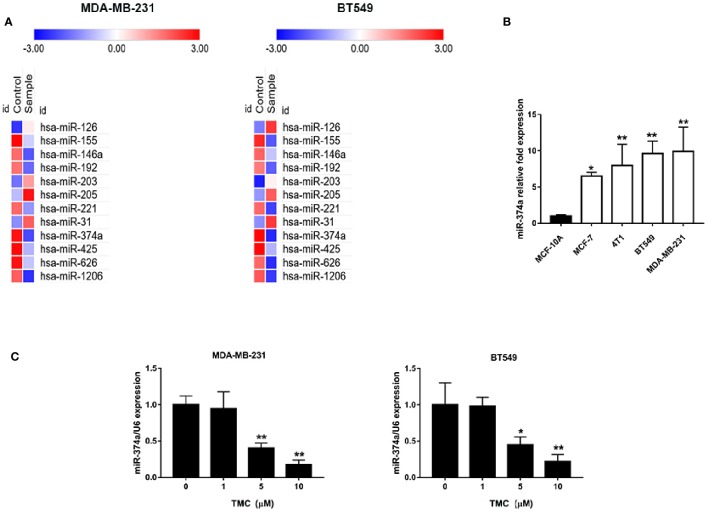
TMC attenuates upregulation of miR-374a in TNBC. **(A)** PCR array analysis of varied miRNA expression in MDA-MB-231 and BT549 cells after exposure to TMC for 24 h. **(B)** Real-time PCR analysis of miR-374a in MCF-10A cells and various types of breast cancers. **(C)** Real-time PCR analysis of miR-374a in MDA-MB-231 and BT549 cells after exposure to TMC for 24 h. Compared with the control group (0 μM), *p < 0.05, **p < 0.01.

We used PCR arrays to determine the changes in miRNAs expression levels (at least 1.5-fold) in TNBC cells after exposure to 10 μM TMC for 24 h. MiR-374a decreased most significantly in all the varied miRNAs after TMC interference. Results displayed that TMC decreased the high expression of miR-374a in both MDA-MB-231 and BT549 cells (**Figure 6A**). Real-time PCR analysis confirmed that TMC significantly inhibited miR-374a in a dose-dependent manner in TNBC cells (*p* < 0.01) (**Figure 6C**). Additionally, miR-374a was expressed at low levels in normal breast cells and high levels in breast cancer cells, especially in TNBC cells (**Figure 6B**). Data from Array Express (https://www.ebi.ac.uk/arrayexpress/) showed that miR-374a was upregulated in TNBC tumor tissues compared to their peritumor breast tissues (**Figure S2**). Taken together, these results indicate that miR-374a is a potential oncogenic miRNA downregulated by TMC.

### TMC Promotes BAX Expression in TNBC Cells Through Downregulation of miR-374a

**Figure 7:**
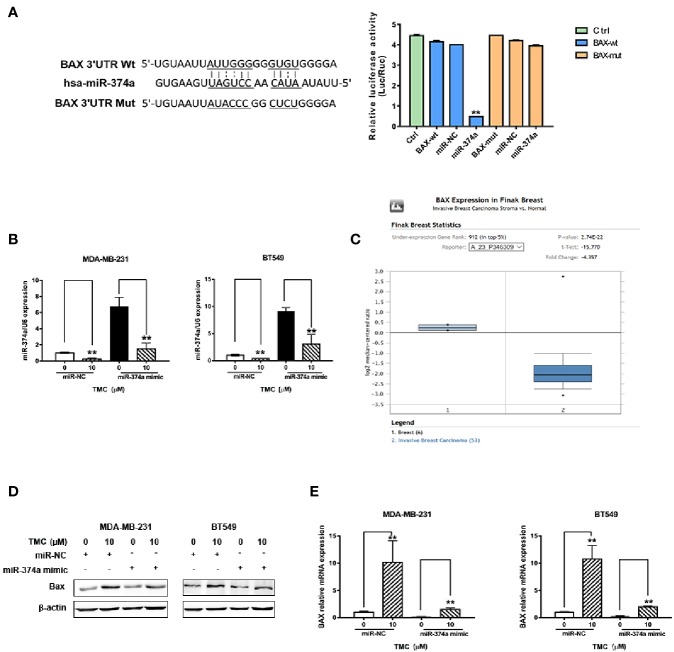
TMC promotes Bax protein and mRNA expression by decreasing miR-374a. **(A)** Dual luciferase reporter assays confirmed the direct binding of miR-374a to the BAX 3′UTR in 293T cells. **(B)** Real-time PCR analysis of miR-200c after exposure to TMC for 24 h following transfection of the miR-374a mimic or miRNA negative control. **(C)** BAX expression in breast cancer tissues and normal breast tissues according to data from the Oncomine database. **(D)** Western blot analysis of Bax after exposure to TMC for 24 h and miR-374a mimic interference. **(E)** Real-time PCR analysis of BAX after exposure to TMC for 24 h and miR-374a mimic interference. Compared with the control group (0 μM), *p < 0.05, **p < 0.01.

Rnahybrid (https://bibiserv.cebitec.uni-bielefeld.de/rnahybrid) predicted the direct interaction of miR-374a and the BAX 3′UTR, suggesting miR-374a as a negative regulator of BAX expression. Luciferase assays confirmed that miR-374a directly binded to the 3′UTR of BAX (**Figure 7A**). Real-time PCR analysis confirmed successful transfection of MDA-MB-231 and BT549 cells with the miR-374a mimic and miRNA negative control (**Figure 7B**). Data from Oncomine (https://www.oncomine.org/) demonstrated that BAX was expressed at low levels, while BCL2 was expressed at high levels in breast cancer tumors compared to normal tissues (**Figures 7C**, **S3**). Western blot results demonstrated that TMC increased Bax protein expression, and that upregulation of miR-374a partly reversed the effect of TMC (**Figure 7D**). Real-time PCR analysis exerted that TMC promoted Bax mRNA expression, and miR-374a upregulation partially blocked the TMC-induced increase in BAX (**Figure 7E**). Taken together, these data suggest that TMC increases Bax protein and mRNA levels through downregulation of miR-374a in TNBC cells.

## Discussion

Breast cancer is a clinically heterogeneous disease, and TNBC is the most aggressive subtype with the worst outcomes (Dent et al., 2007). Although there have been advancements in the multidisciplinary treatments available, therapeutic options for advanced TNBC remain limited (Isakoff, 2010; Wahba and El-Hadaad, 2015). The search for innovative and effective drug candidates becomes essential for the improvement of current TNBC treatments. Recently, research attention has shifted towards Chinese medicine, which is considered to contain compounds with potential anticancer properties. The active ingredients isolated from Chinese herbs have gradually been developed as novel therapeutic agents (Peng et al., 2019a). Interestingly, we previously identified a natural flavonoid, ISL, with cytotoxic effects on MDA-MB-231 proliferation. We synthesized an ISL derivative, TMC, with a stronger inhibitory effect on TNBC cell growth (Peng et al., 2016a). In the present study, we first found that TMC inhibited TNBC cell foci formation, migration, and invasion. Additionally, TMC suppressed tumor growth, indicating that TMC should be considered as a potential drug candidate for further investigation.

Apoptosis is a strictly organized process with the ability to attenuate tumor progression (Abotaleb et al., 2019). There are two major pathways involved in apoptosis: the extrinsic pathway and the intrinsic pathway. In the extrinsic pathway, binding of death signals to trimeric death ligands is an important mediator that activates signal transduction cascades (Igney and Krammer, 2002). In the intrinsic pathway, activated Bax and Bak mediate an increase in mitochondrial stress (Indran et al., 2011). Inhibiting anti-apoptotic Bcl-xL and Bcl-2 activates Bax and Bak (Fan et al., 2005). Our study found that TMC induced apoptosis in a dose-dependent manner in TNBC cells, and TMC affected proapoptotic Bax and anti-apoptotic Bcl-2, suggesting a mediator role for TMC in the intrinsic pathway. Additionally, TMC increased Bax mRNA expression, suggesting TMC with the capacity to modulate BAX levels either transcriptionally or post-transcriptionally.

BAX belongs to the BCL-2 protein family, and its activation drives mitochondria-dependent apoptosis. BH3-only members, such as BID and BAD, can directly engage BAX and convert it into an oligomerization form on the mitochondrial outer membrane, triggering apoptosis. Recent studies exerted that BAX activation was also affected by post-translational modifications, membrane anchors and the autonomous retro-translocation of BAX to the cytosol (Adams, 2019). We firstly reported that miR-374a directly binded the 3′UTR of BAX, negatively regulating Bax mRNA and protein levels. These results displayed that miR-374a affected apoptosis in TNBC cells in an oncogenic manner to promote tumor initiation. MiR-374a expression was increased by about 3.5-fold in TNBC tissue compared to adjacent normal tissues, which was associated with overall worse survival rates. In addition, miR-374a promoted cell survival, proliferation, and migration in TNBC cells, and induced tumor progression *in vivo* (Son et al., 2019). Our results confirmed the upregulation of miR-374a in various TNBC cell lines, including MDA-MB-231, 4T1, and BT549 cells. Our results also manifested that miR-374a promoted TNBC proliferation through the inhibition of apoptosis. These findings suggest TMC is a potential TNBC therapeutic target. Our study identified that TMC significantly inhibited miR-374a in TNBC cells, and upregulation of miR-374a partially reversed its effects on Bax mRNA and protein expression, indicating that TMC as a potent inhibitor of miR-374a in TNBC cells.

## Conclusion

TNBC is a subtype of breast cancer without rapid and efficient therapies. TMC exerts anti-TNBC effects on cell proliferation and invasion, suggesting that TMC is a potential drug candidate for TNBC treatment. We also detect that TMC significantly downregulates miR-374a expression, and TMC increases Bax protein and mRNA levels through miR-374a downregulation. Luciferase assays confirms the direct interaction between miR-374a and the 3′UTR of BAX. Accordingly, we show that miR-374a is a negative regulator of Bax, affecting apoptosis in TNBC cells. TMC also represses TNBC tumor growth *in vivo*. Thus, this is the first report demonstrating that TMC inhibits tumor initiation and progression by modulating the miR-374a/BAX axis in TNBC.

## Data Availability Statement

The raw data supporting the conclusions of this article will be made available by the authors, without undue reservation, to any qualified researcher. Datasets generated for this article can be found in [Array express] using the accession number E-GEOD-40525.

## Ethics Statement

The animal study was reviewed and approved by corresponding committees in Sichuan University and Chengdu University of Traditional Chinese Medicine according to the institutional standard guidelines.

## Author Contributions

The study was designed by FP and CP. The manuscript was written by FP and LX. FP performed experiments and analyzed data with LX, XX, and RH. RH, XX, and HT revised the manuscript. CP supported the study.

## Funding

The study was supported by the Fundamental Research Funds for the Central Universities (no. YJ201880), the National Natural Science Foundation of China (no. 81630101; no. 81891012), the Key Project of Science and Technology Department of Sichuan Province (no. 20ZDYF3092), and the Open Research Fund of Chengdu University of Traditional Chinese Medicine Key Laboratory of Systematic Research of Distinctive Chinese Medicine Resources in Southwest China.

## Conflict of Interest

The authors declare that the research was conducted in the absence of any commercial or financial relationships that could be construed as a potential conflict of interest.
