# Supplementary material for: Isoliquiritigenin Derivative Regulates miR-374a/BAX Axis to Suppress Triple-Negative Breast Cancer Tumorigenesis and Development
Source: Front Pharmacol. 2020 Mar 31;11:378. doi: 10.3389/fphar.2020.00378 (PMC7137655; doi:10.3389/fphar.2020.00378)
Supplement: Supplementary file 1 [file DataSheet_2.pdf]

# Isoliquiritigenin derivative regulates miR-374a/BAX axis to suppress triple negative breast cancer tumorigenesis and development

Fu Peng[1,2,3], Liang Xiong[2], Xiaofang Xie[2], Hailin Tang[4], Ruizhen Huang[3*], Cheng Peng[2*]

*1. Key Laboratory of Drug-Targeting and Drug Delivery System of the Education Ministry and Sichuan Province, West China School of Pharmacy, Sichuan University, Chengdu, China;*

*2. Key Laboratory of Systematic Research of Distinctive Chinese Medicine Resources in Southwest China, Chengdu University of Traditional Chinese Medicine, Chengdu, China;*

*3. Cardiovascular Department, Hospital of Chengdu University of Traditional Chinese Medicine, Chengdu, China*

*4. Department of Breast Oncology, Sun Yat-Sen University Cancer Center, State Key Laboratory of Oncology in South China, Guangzhou, Guangdong, China*

*Correspondence: Cheng Peng, Tel:+86-028-6180-0018, Email:pengchengchengdu@126.com; Ruizhen Huang. Tel:+86-028-8778-3481, Email:HuangRuizhen41@foxmail.com

## Table S1. The chemical information of TMA

| Chemical name | Formula | Type of compound | Molecular weight (g/mol) |
|---|---|---|---|
| 3′,4′,5′,4″-Tetramethoxychalcone | $C_{19}H_{20}O$ | Chalcones | 264.36 |

| | |
|---|---|
| **BAX** | (F) CCGCCGTGGACACAGACT |
| | (R) TTGAAGTTGCCGTCAGAAAACA |
| **GAPDH** | (F) GACTCATGACCACAGTCCATGC |
| | (R) AGAGGCAGGGATGATGTTCTG |
| **U6** | (F) CTCGCTTCGGCAGCACA |
| | (R) AACGCTTCACGAATTTGCGT |

**Table S2. The primers used for Real-time PCR**

| | |
|---|---|
| **BAX wide-type** | (F) CGGAGCGTCCTGGCCGAGTCACTGAAGCG |
| | (R) CGAAGCTTCCTAACCCACAGACCCCACA |
| **BAX mut** | (F) CTCTTCCCCACACCCGGGTTACGTTACAAAAGTAAGAAAATG |
| | (R) GGGTGTGGGGAAGAGTGGTC |

**Table S3. The primers used for colony PCR**

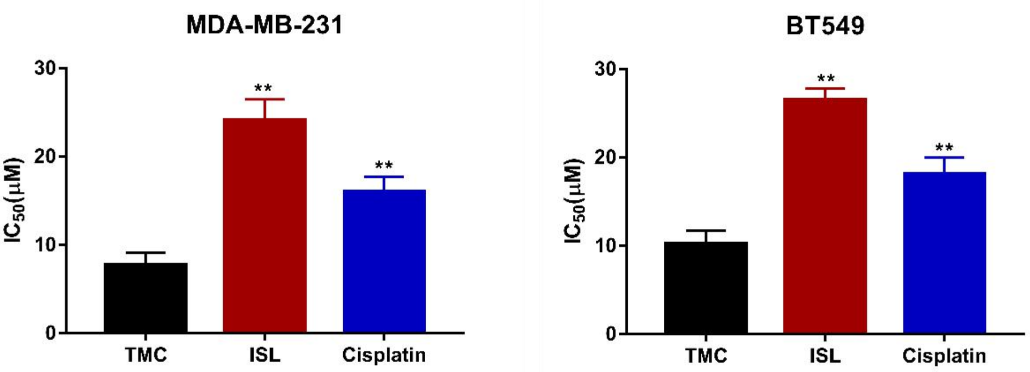


**Fig S1.** The $IC_{50}$ values of MDA-MB-231 and BT549 cells with the treatment of TMC, ISL and cisplatin for 24 h (**$p < 0.01$, compared to TMC group).

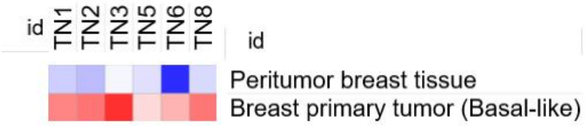


**Fig S2.** MiR-374a was highly expressed in TNBC compared to peritumor breast tissues according to data from array express (https://www.ebi.ac.uk/arrayexpress/experiments/E-GEOD-40525/) analyzed by morpheus.

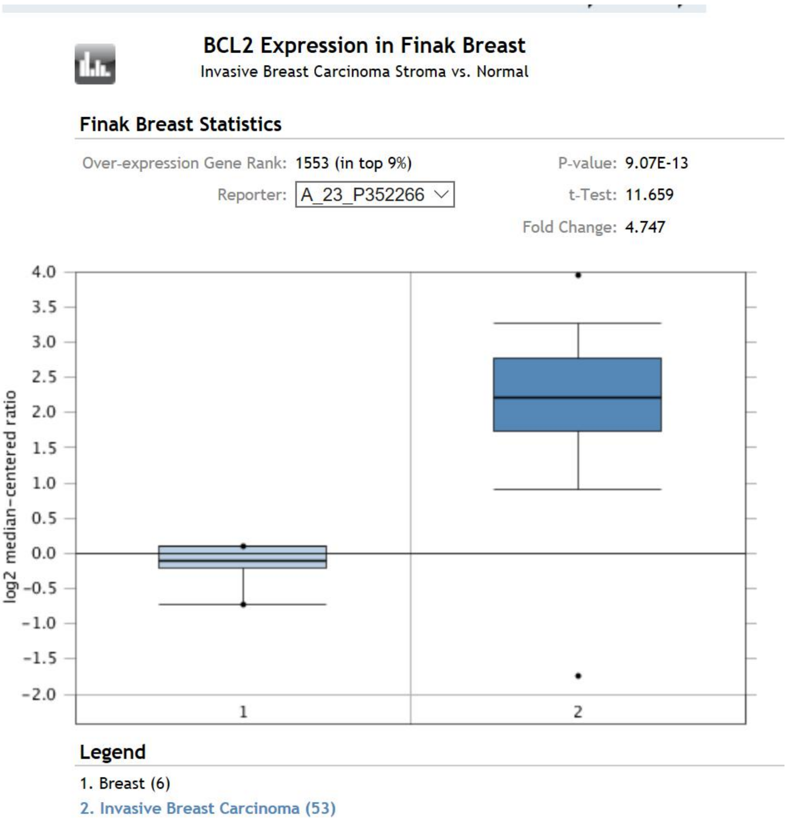


**Fig S3.** BCL2 was highly expressed in invasive breast cancer tissues compared to non-tumor tissues according to data from oncomine (https://www.oncomine.org/resource/main.html).
