# Supplementary figures and images for: Isoliquiritigenin Derivative Regulates miR-374a/BAX Axis to Suppress Triple-Negative Breast Cancer Tumorigenesis and Development
Source: Front Pharmacol. 2020 Mar 31;11:378. doi: 10.3389/fphar.2020.00378 (PMC7137655; doi:10.3389/fphar.2020.00378)

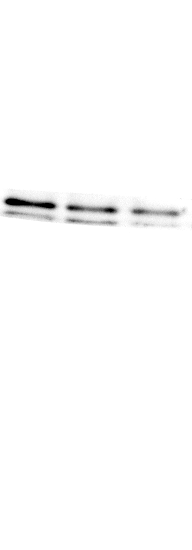

Supplement: Supplementary file 2 [file DataSheet_1.zip › Figure 5E/bcl-2.tif]

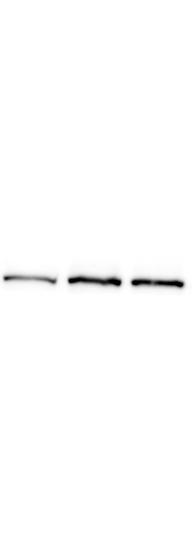

Supplement: Supplementary file 2 [file DataSheet_1.zip › Figure 5E/bax.tif]

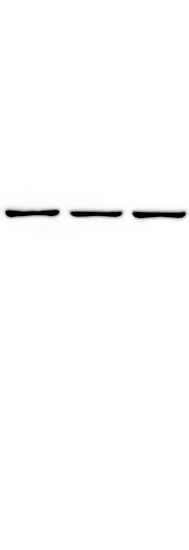

Supplement: Supplementary file 2 [file DataSheet_1.zip › Figure 5E/actin.tif]

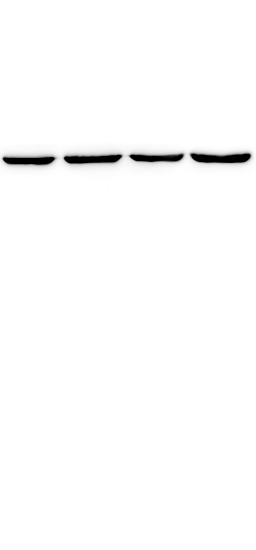

Supplement: Supplementary file 2 [file DataSheet_1.zip › Figure 7E/BT549/actin.tif]

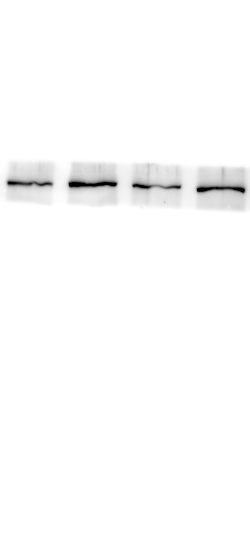

Supplement: Supplementary file 2 [file DataSheet_1.zip › Figure 7E/BT549/Bax.tif]

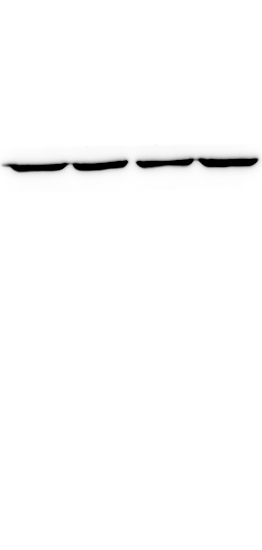

Supplement: Supplementary file 2 [file DataSheet_1.zip › Figure 7E/MDA-MB-231/actin.tif]

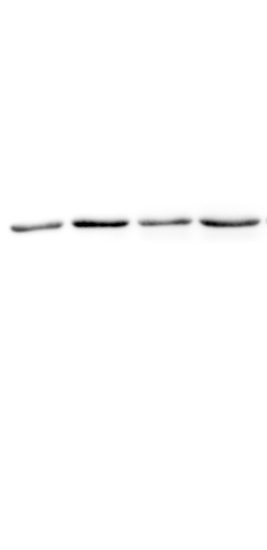

Supplement: Supplementary file 2 [file DataSheet_1.zip › Figure 7E/MDA-MB-231/Bax.tif]

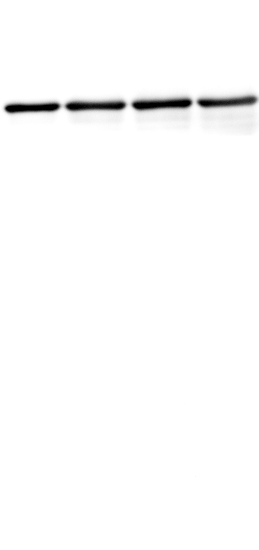

Supplement: Supplementary file 2 [file DataSheet_1.zip › Figure 4C/BT549/actin.tif]

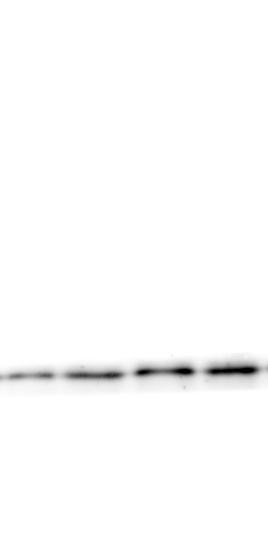

Supplement: Supplementary file 2 [file DataSheet_1.zip › Figure 4C/BT549/bax.tif]

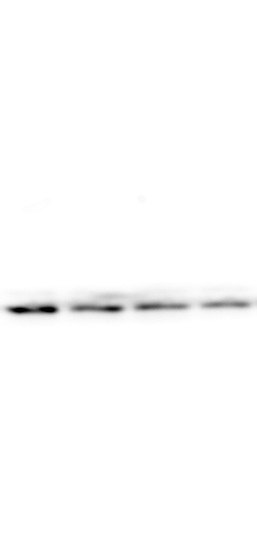

Supplement: Supplementary file 2 [file DataSheet_1.zip › Figure 4C/BT549/bcl-2.tif]

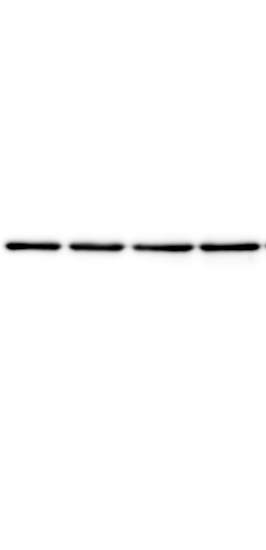

Supplement: Supplementary file 2 [file DataSheet_1.zip › Figure 4C/MDA-MB-231/actin.tif]

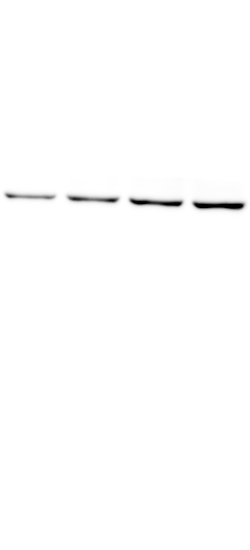

Supplement: Supplementary file 2 [file DataSheet_1.zip › Figure 4C/MDA-MB-231/bax.tif]

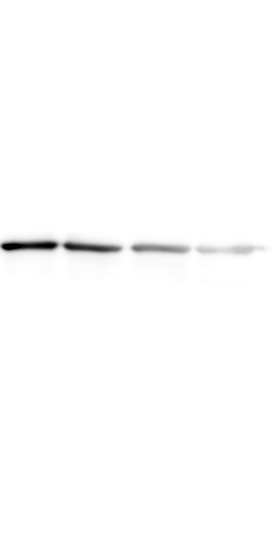

Supplement: Supplementary file 2 [file DataSheet_1.zip › Figure 4C/MDA-MB-231/bcl-2.tif]
